# Flat hardness distribution in AA6061 joints by linear friction welding

**DOI:** 10.1038/s41598-021-91249-5

**Published:** 2021-06-03

**Authors:** Jeong-Won Choi, Weihao Li, Kohsaku Ushioda, Hidetoshi Fujii

**Affiliations:** Joining and Welding Research Institute, Osaka University, Osaka, Ibaraki 567-0047 Japan

**Keywords:** Metals and alloys, Structural materials, Mechanical properties

## Abstract

It is known that one of the main concerns associated with the conventional welding of precipitation-strengthened Al alloys is the formation of softening regions, resulting in the deterioration of mechanical properties. In this study, we show that linear friction welding (LFW) can completely suppress softening regions in precipitation-strengthened AA6061-T6 alloy by introducing a large shear strain and by controlling the interfacial temperature. We found that the LFW process resulted in an extremely low interfacial temperature; it decreased as the applied pressure increased from 50 to 240 MPa. This approach can essentially suppress both softening and hardening regions, leading to uniform hardness distribution in Al joints. The high-pressure LFW process demonstrated here can thus provide an innovated guidance to obtain high-performance Al alloy joints and be extended to other precipitation-strengthened Al alloys, which undergo high-temperature softening.

## Introduction

Lightweight structural materials are necessary to reduce fuel consumption and emissions from vehicles^1^. The use of lightweight Al alloys in vehicles has increased rapidly over the past few decades due to their high specific strength and high corrosion resistance. Vehicle weight can be successfully reduced by partially replacing steel with Al alloys. Among Al alloys, precipitation-strengthened Al-Mg-Si alloys have gained much attention due to their superior mechanical properties^2–5^. However, one of the main concerns associated with the conventional welding of these alloys is the formation of softening regions at high temperatures^6^. In addition, from a metallurgical perspective, melting and solidification bring coarse grains together; further, significant softening occurs in the heat-affected zone (HAZ) due to the dissolution of precipitates contributing to the strengthening of the base materials, which degrade the mechanical properties of the joint^7^ and limit their widespread applications. To address these problems, friction stir welding (FSW) was performed to join Al6061 alloys^8,9^. Lim et al. reported that the joint efficiency for tensile properties was only approximately 80% due to the softening occurring in the HAZ regions^8^. Liu et al. successfully fabricated sound Al6061 joints without the formation of welding defects^9^. Nevertheless, the peak tensile strength was found to be equivalent to only 69% to that of base material and all the joints fractured at the weakened HAZ regions.

Here we show that LFW can effectively suppress the softening of the HAZ in precipitation-strengthened Al alloys by controlling the temperature using the applied pressure^10,11^. In the present study, we show that extremely high applied pressures of LFW result in extremely low-temperature welding of the AA6061 alloy. This approach can be expected to essentially suppress the softening of the HAZ in Al-Mg-Si alloys. Moreover, the obtained joints provide highly refined grains with an average diameter less than 1 μm at the weld centre of the joints due to the highly rapid heating and cooling as well as the large induced shear strain. This high-applied-pressure joining method can be extended to other precipitation-strengthened Al alloys such as the 2xxx series and 7xxx series, which are sensitive to high-temperature softening.

## Results and discussion

### Hardness distribution

**Figure 1:**
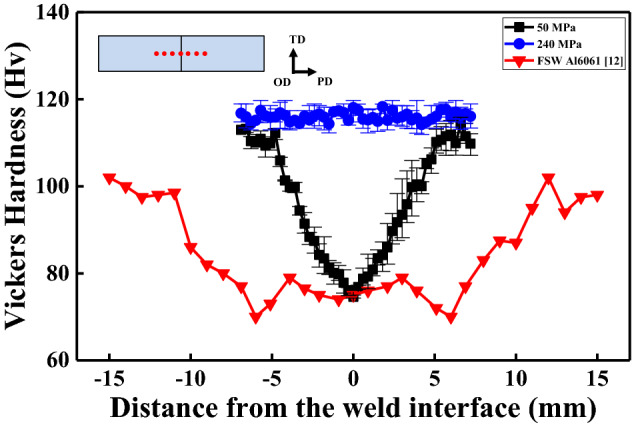
Hardness profiles of Al6061-T6 joints obtained at applied pressures of 50 MPa and 240 MPa and FSW Al6061-T6 joints. TD, OD, and PD stand for transverse direction, oscillation direction, and pressure direction, respectively.

Figure 1 shows the Vickers hardness profiles of AA6061 joints as a function of the distance from the weld centre line; the joints were fabricated at pressures of 50 and 240 MPa, as well as by conventional FSW^12^. The hardness distributions were found to be significantly dependent on the applied pressure^13^. It was noteworthy that the formation of softening regions was completely suppressed in LFW joints obtained at high applied pressures (240 MPa). The hardness at the weld centre increased from 74 to 119 Hv as the applied pressure increased from 50 to 240 MPa. No softening regions were observed and the hardness profile was flat in joints obtained at 240 MPa; these joints exhibited a relatively low interfacial temperature. Meanwhile, wide softening regions were observed in FSW joints^12^ and LFW joints fabricated at 50 MPa, attributed to a high interfacial temperature in these cases.

### Joint microstructure

**Figure 2:**
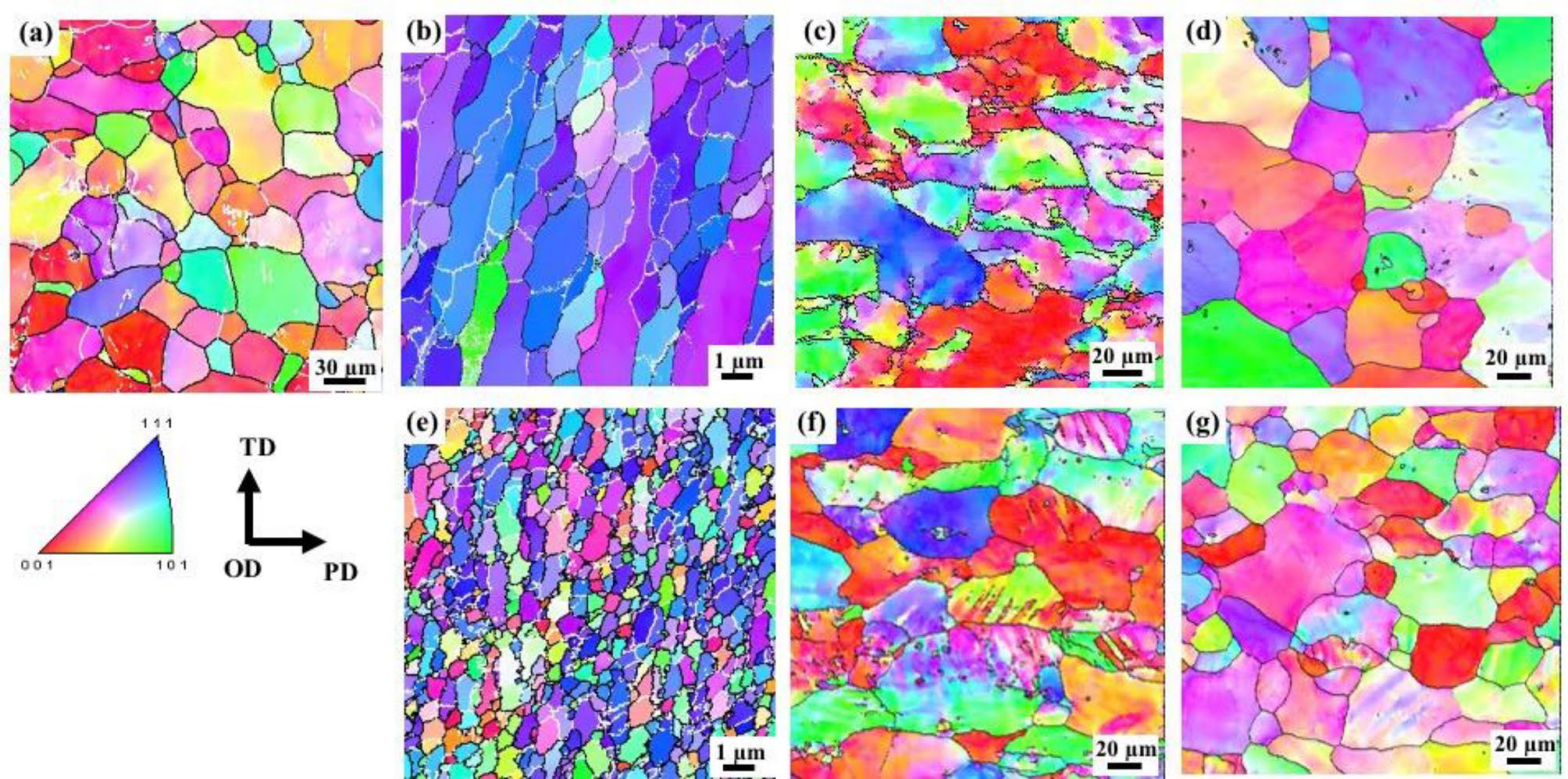
EBSD maps. (**a**) Base material. Joint obtained at 50 MPa—(**b**) weld centre, (**c**) 0.5 mm from the weld centre, and (**d**) 2 mm from the weld centre. Joint obtained at 240 MPa—(**e**) weld centre, (**f**) 0.5 mm from the weld centre, and (**g**) 2 mm from the weld centre. The black and white lines represent HAGBs and LAGBs, respectively. TD, OD, and PD indicate transverse, oscillation, and pressure directions, respectively.

Figure 2 shows the electron backscatter diffraction (EBSD) inverse pole figure maps of LFW joints obtained at 50 and 240 MPa, and the base material. The observed regions were the weld interface and regions 0.5 and 2 mm away from the weld centre. The base material consisted of coarse equiaxed grains with an average diameter of ~ 45 μm. LFW resulted in a significantly refined microstructure at the weld centre (Fig. 2b,e) as the workpieces underwent severe plastic deformation, dynamic restoration, and rapid cooling. The average diameter of the equiaxed grains formed at the weld centre decreased from 2.4 to 0.3 μm as the applied pressure increased from 50 to 240 MPa. This difference is attributed to dynamic recrystallisation (DRX) at reduced interfacial temperatures (such as that at 240 MPa) during LFW^10,11^. At 50 MPa, the equiaxed grains at the weld centre were coarser than those formed at 240 MPa. Furthermore, the grains were completely flattened in the shear direction by plastic deformation; these refined and recrystallised grains predominantly exhibited a <111> orientation. These results suggest that continuous dynamic recrystallisation (CDRX) occurred at the weld centre of the joint obtained at 50 MPa due to a high interfacial temperature^14^. Meanwhile, the grains formed at 240 MPa were extremely refined and exhibited a randomly distributed texture, probably due to nucleation in different orientations. The fraction of high-angle grain boundaries (HAGBs) increased, even though there existed some low-angle grain boundaries (LAGBs), as shown in Fig. 2e. Based on these observations, it can be stated that discontinuous dynamic recrystallisation (DDRX) occurred at the weld centre of joints fabricated at 240 MPa due to a low interfacial temperature. Meanwhile, the grains formed at 0.5 and 2 mm away from the weld interface (Fig. 2c,d,f,g) were much coarser (~ 35 μm) than those formed at the weld centre (Fig. 2b,e). There were no significant differences in the joint microstructures in regions 0.5 and 2 mm away from the weld centre, even though the applied pressure varied (the hardness values were significantly different); this phenomenon will be discussed later in this report.

**Figure 3:**
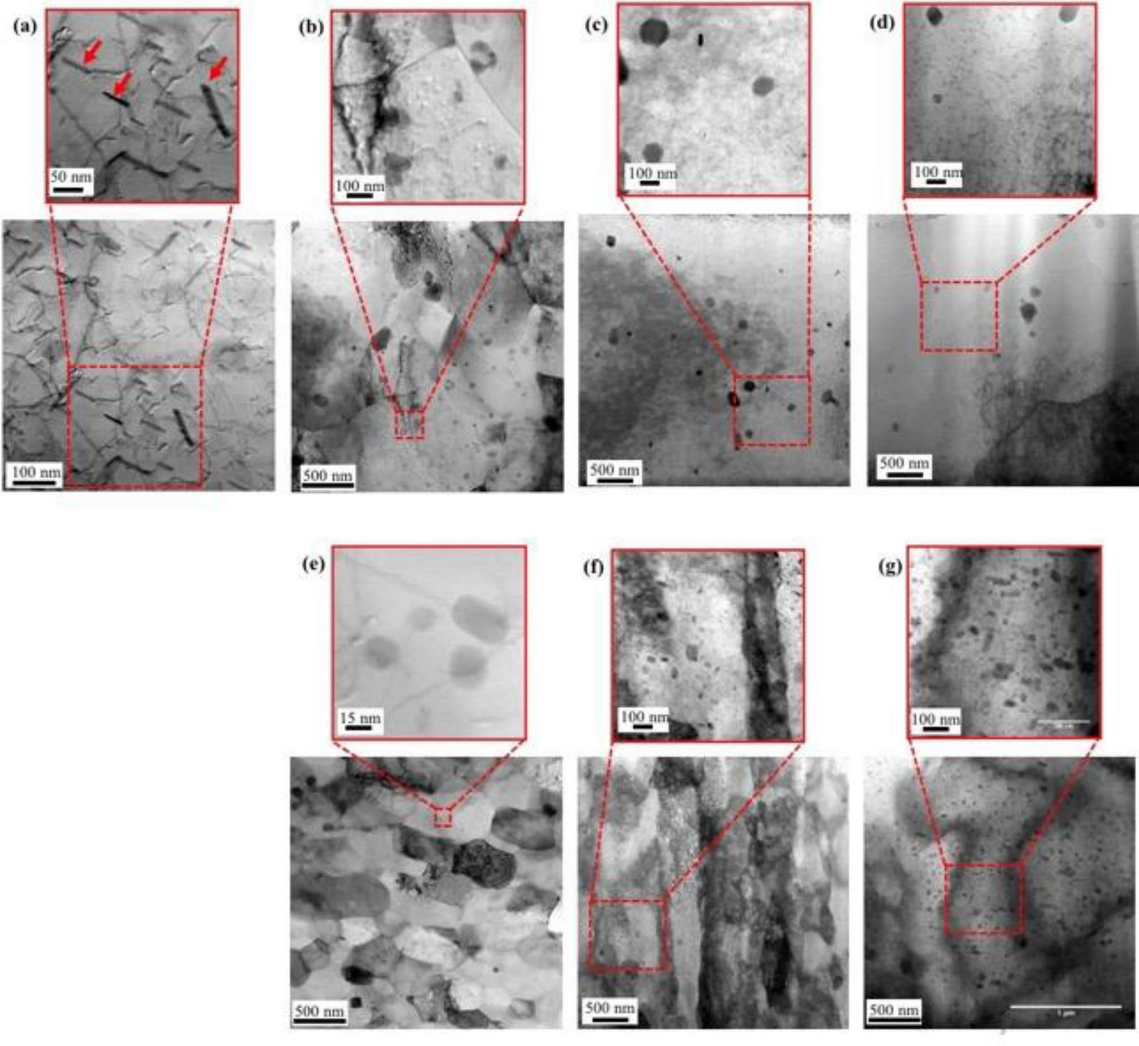
TEM images. (**a**) Base material. Joint obtained at 50 MPa—(**b**) weld centre, (**c**) 0.5 mm from the weld centre, and (**d**) 2 mm from the weld centre. Joint obtained at 240 MPa—(**e**) weld centre, (**f**) 0.5 mm from the weld centre, and (**g**) 2 mm from the weld centre.

Precipitation strengthening is the most dominant strengthening mechanism in AA6061 alloys; hence, it is necessary to examine precipitates in terms of their size, shape, distribution density, and chemical composition^15^. To examine the microstructure of joints obtained at different applied pressures, the cross-sections of the fabricated joints and base material were examined by transmission electron microscopy (TEM). Figure 3a shows the microstructure of the base material. Needle-shaped precipitates of Mg_5_Si_6_, which are typically found in low-temperature aged Al-Mg-Si^16^, were observed in the AA6061 alloys, as indicated by the red arrows. Figure 3b–g show the microstructures at the weld centre and regions 0.5 and 2 mm away from the weld centre in joints obtained at 50 and 240 MPa. Clearly, extremely refined equiaxed grains were formed at the weld centre of joints obtained at 240 MPa (Fig. 3e). Moreover, needle-shaped precipitates in the base material were transformed into spherical precipitates during LFW. This phenomenon may be explained as follows. The precipitates formed in the base material may have broken down into finer fragments with a spheroidised shape^17^ at the weld centre of the LFW joints. Moreover, it can also be considered that the precipitates underwent dissolution at high temperatures during LFW, followed by re-precipitation during cooling (Fig. 3b), similar to that in FSW^18–20^. While the average diameter of the precipitates decreased, their volume fraction increased with increasing applied pressure due to a decrease in the interfacial temperature. A larger amount of precipitates dissolved at higher temperatures, rather than at lower temperatures, and grew during cooling. Similarly, a larger amount of precipitates could be observed at regions 0.5 and 2 mm away from the weld centre than at the weld centre. The dislocation density, evaluated from the TEM images (Fig. 3b–e,g), is relatively low, i.e., 4 × 10^13^ m^−2^ for 240 MPa—Centre and 1 × 10^14^ m^−2^ for 240 MPa–0.5 mm as illustrated in Supplementary Information (Table 1). However, the joints obtained at 240 MPa, particularly at the region closer to the weld centre (0.5 mm), seem to be subjected to higher strain at low temperature during LFW, which resulted in increased dislocation density (Fig. 3f). Nevertheless, there were no significant differences in the diameter and volume fraction of the precipitates formed at distances of 0.5 and 2 mm from the weld centre, irrespective of the applied pressure.

### Performance of LFW joints

**Figure 4:**
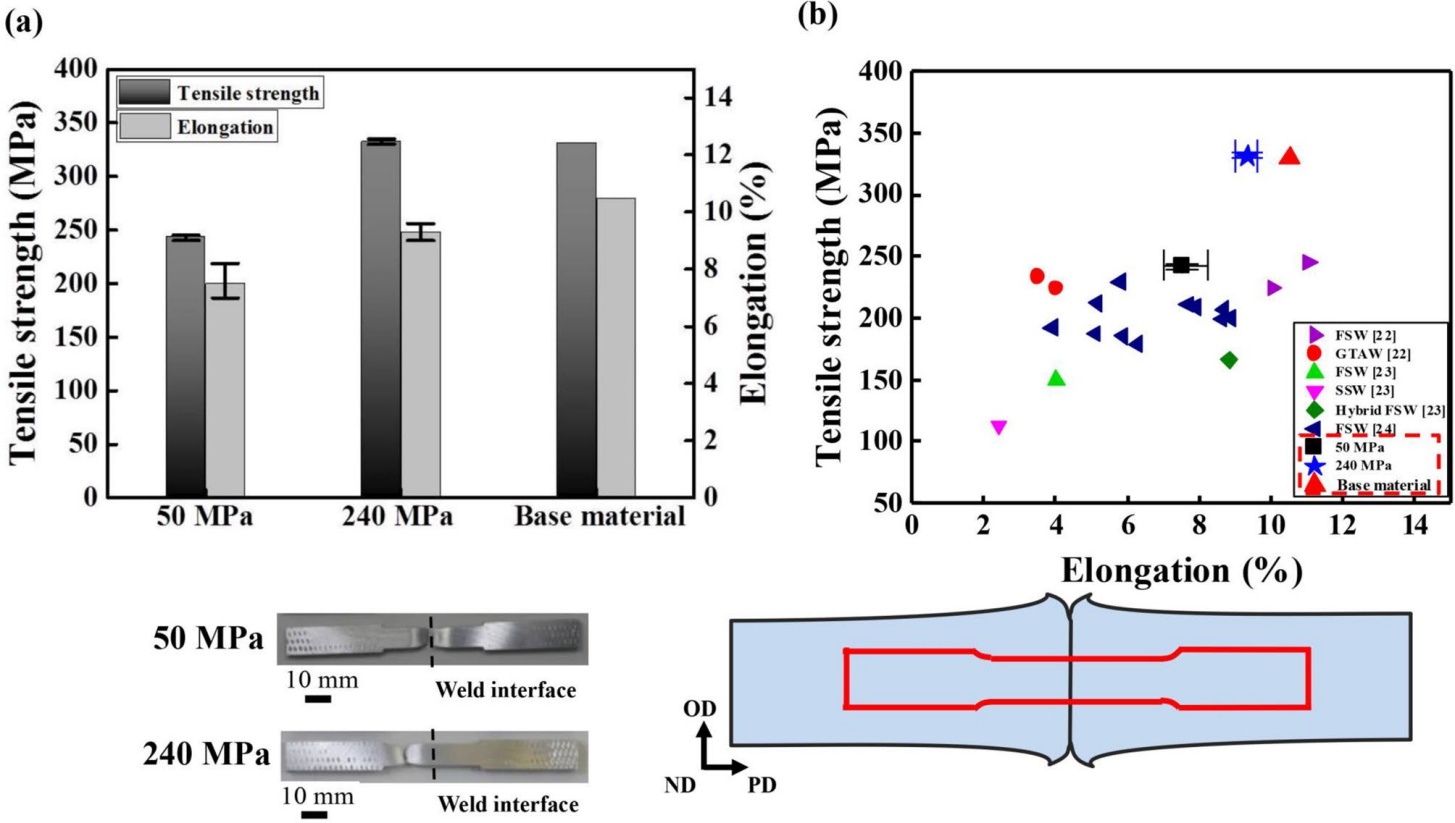
(**a**) Stress strain curves of base material and the joints obtained at different applied pressure of 50 MPa and 240 MPa and (**b**) relationship between tensile strength and elongation of the weld joints obtained by various welding methods. The results obtained in the present study are indicated by red dotted lines.

To evaluate the mechanical properties of the fabricated LFW AA6061 joints, tensile tests were conducted. The testing direction was perpendicular to the welding line of joints fabricated at different applied pressures (Fig. 4). At 50 MPa, the joint-strength efficiency was only 82% and was accompanied by poor ductility due to the existence of softened regions^21^. Meanwhile, at 240 MPa, the joints exhibited excellent tensile properties, which were almost equivalent to those of the base material (joint efficiency of 100%), with only a slight reduction in ductility (90% of the ductility of the base material). The tensile specimens of joints obtained at 50 MPa fractured near the weld interface, while the joints obtained at 240 MPa fractured in the base material.

The weld joints of precipitation-strengthened AA6061 alloys obtained by conventional methods, such as gas tungsten arc welding (GTAW)^22^, FSW^22–24^, and hybrid FSW^23^, suffer from low tensile strength and poor ductility (Fig. 4b). In contrast, the tensile strength of joints obtained by high-pressure LFW was almost equal to that of the base material (Fig. 4b). It is indeed surprising to achieve such high-strength AA6061 joints and to the best of our knowledge, this is the first time that such materials have been reported. Thus, it may be stated that high-pressure LFW can be used to solve the problems encountered in the welding of precipitation-strengthened Al alloys. In Al-Mg-Si alloys, softening occurs as the precipitates dissolve at a higher temperature of 200 °C considering AA6061, which results in the deterioration of mechanical properties^25^. High-pressure LFW enables welding below or near this temperature so that the formation of both, softening and hardening regions, can be effectively suppressed.

### Strengthening mechanism in the joints

A uniform hardness distribution was observed in joints fabricated at 240 MPa, even though the microstructures with respect to grain diameter, precipitates, and dislocations were different depending on the region. To comprehensively understand these results, the strengthening mechanism of the joints was further investigated. It is considered that the mechanical properties of LFW AA6061 joints are associated with precipitation strengthening, dislocation strengthening, and grain-boundary strengthening, as described in Eq. (1),

1 $$\sigma  = {\sigma _0} + {\sigma _{{\text{ppt}}}} + {\sigma _{{\text{disl}}}} + {k_y}{D^{{{ - 1}/2}}}$$

where $\sigma$ is the yield strength of the joint,${\sigma }_{0}$ is a constant, *σ*_ppt_ represents precipitation strengthening, σ_disl_ represents dislocation strengthening, and ${k}_{y}$ and $D$ are a constant and the average grain diameter respectively. The grain boundary strengthening is calculated using the Hall–Petch relationship assuming HAGBs^26,27^. Furthermore, the formation of LAGBs (white lines, Fig. 2), presumably due to dislocation rearrangement during LFW, was considered as another boundary-strengthening mechanism as proposed by Bowen et al.^28^.

We employed the Ashby-Orowan model^29^ to understand precipitation strengthening and the Bailey equation for dislocation strengthening. The yield strength described in Eq. (1) can be expressed as per Eq. (2),

2 $$\sigma = {\sigma }_{0} + \frac{0.4\text{Gb}}{\pi {\left(1-v\right)}^{1/2}}\frac{\ln \left( {{\raise0.7ex\hbox{${\overline d }$} \!\mathord{\left/
 {\vphantom {{\overline d } b}}\right.\kern-\nulldelimiterspace}
\!\lower0.7ex\hbox{$b$}}} \right)}{\overline{\lambda }} + aMGb\sqrt{\rho } +{k}_{y}{D}^{-\frac{1}{2}} +\beta MGb\sqrt{\frac{1.5{S}_{v}\theta }{b}}$$

where G is the shear modulus (26 GPa), $b$ is the Burgers vector (0.286 nm), $v$ is the Poisson’s ratio (0.33), and $\overline{d}$ and $\overline{\lambda }$ are the size and interval of the precipitates, respectively (these parameters will be defined later in this section). Furthermore, $a$ is a constant (0.5)$, M$ is the Taylor factor (3.06), and $\rho$ is the dislocation density, given by the total length of the dislocations divided by the total specimen volume. For AA6061 alloys, ${\sigma }_{0}$ and ${k}_{y}$ were considered to be 50 MPa and 70 MPa ${\mu m}^{1/2}$, respectively^30^. Meanwhile, ${S}_{v}$ is the LAGB length per unit area, $\theta$ is the average misorientation of LAGBs, and $\beta$ = 0.1^31^. $\overline{d}$ and $\overline{\lambda }$ were defined using the average diameter ($d$) and volume fraction ($f$) of the precipitates, respectively, as follows:^29^

3 $$\overline d  = \sqrt {\frac{2}{3}} d$$

4 $$\overline \lambda   = \overline d \left( {\sqrt {\frac{\pi }{{4f}}}  - 1} \right)$$

The calculated values for each strengthening mechanism, together with the necessary parameters for predicting the performance of LFW AA6061 joints produced at different applied pressures and the base material, are listed in Supplementary Information (Tables 1 and 2). From the results, it could be inferred that the contribution of strengthening mechanisms was different in the base material and high-pressure LFW AA6061 joints both at the weld centre and at distances of 0.5 and 2 mm from the weld centre, even though the hardness values were similar (Fig. 1). Precipitation strengthening was the most effective mechanism in the base material, with grain-boundary and dislocation strengthening having only a minimal effect. Nevertheless, at the weld centre, LFW drastically reduced the effect of precipitation strengthening as the precipitates that formed in the base material dissolved. In contrast, the effect of grain-boundary strengthening increased as workpieces with extremely severe plastic deformation dynamically recrystallised at relatively low temperatures, leading to the formation of ultra-fine grains (~ 300 nm) during LFW (Fig. 3e). The contribution of dislocation strengthening at the weld centre of the LFW joints was not high, irrespective of the applied stress. DDRX was presumed to result in numerous HAGBs when LFW was carried out at high pressures (Fig. 2e). At distances of 0.5 and 2 mm from the weld centre, precipitation strengthening and dislocation strengthening were the dominant mechanisms followed by grain-boundary strengthening. However, LAGBs are also expected to have a considerable effect. The actual contribution of the strengthening mechanism is presumably dependent on the distance from the weld centre. However, the hardness profile was surprisingly flat despite the significant changes in microstructure in different regions of the joints. The absence of both softened and hardened regions in the joints can enable superior tensile properties, equivalent to those of the base material. Thus, high-pressure LFW can yield sound joints with superior mechanical properties, enabling the welding of precipitation-hardened Al alloys as well as other metallic alloys.

Based on the reported results, the influence of the applied pressure on the hardness distribution, microstructure and the mechanical properties of the welded joints is discussed as follows. In the joint fabricated at 50 MPa, since the coarser equiaxed grains, a relatively lower dislocation density and volume fraction of the precipitate due to the high interfacial temperature during LFW are formed near the weld centre, the weld centre is weaker than the base material in the joint, thus it fractured near the weld interface and the mechanical properties of the joint are deteriorated. In the joint fabricated at 240 MPa, since the joint has the refined equiaxed grains, a relatively higher dislocation density and volume fraction of the precipitate due to the low interfacial temperature during LFW are formed near the weld centre, no softening region are formed in the joint. Furthermore, the formation of the hardening region was also suppressed due to the appropriate interfacial temperature, thus it fractured at the base material and has the flat hardness distribution, which results in the equivalent mechanical properties to the base material.

## Methods

### LFW process

AA6061-T6 alloy specimens (5 mm (thickness) × 25 mm (width) × 65 mm (length)) were used for LFW processing in the present study. The chemical compositions of the base material are listed in Supplementary Information (Table 3). The width direction was equivalent to the direction of linear motion and the weld interface corresponded to thickness-width plane. In order to investigate the influence of interfacial temperature on joint quality, the LFW process was carried out at different applied pressures of 50 and 240 MPa at a constant frequency and amplitude of 25 Hz and 2 mm, respectively as listed in Supplementary Information (Table 4). The LFW process was carried out using metallic jig with a length of 60 mm, consequently the protruding length of the sample was 5 mm on both sides. The total upset was set as 5 mm, and when it was achieved, the welding process was automatically completed.

### Microstructure observation

The cross-sectional microstructure of the obtained joints was observed by using a JEOL JSM-7001FA scanning electron microscope (SEM) with EBSD and JEOL JEM-2100F TEM. The specimens necessary for this examination were prepared by electric discharge machining, followed by mechanical polishing with abrasive papers up to 4000 grit and final polishing with 3- and 1-μm diamond suspensions. Subsequently, EBSD specimens were subjected to electronic polishing with a solution of 40 mL HClO_4_ + 160 mL C_2_H_5_OH for 15 s at 20 V and 0 °C before examination. For EBSD observations, the SEM was operated at 15 kV using the TSL OIM™ software; the step size was set at 0.07 μm. TEM specimens (150 nm thick) were prepared by JEOL JIB-4500 focused ion beam (FIB) milling. Precipitate size and fraction were approximately evaluated along with dislocation density using TEM operating at 200 kV. The total length of the dislocations was calculated by Image J software and it was subsequently divided by the total volume to evaluate dislocation density.

### Evaluation of mechanical properties

The Vickers hardness of LFW joints fabricated at different applied pressures was measured as a function of distance from the weld interface at a load of 0.98 N with a dwell time of 15 s using a microhardness test machine (FUTURE-TECH FM-300). Hardness was measured at 0.3-mm intervals along the direction perpendicular to the weld centre of the joints. Specimens for tensile testing (25 mm (gauge length) × 6 mm (gauge width) × 4.5 mm (gauge thickness)) were prepared according to ASTM E8 standard using an electric discharge machine, in a direction perpendicular to the weld centre line. Then, the tensile tests were conducted at a cross-head speed of 1 mm/min using a tensile test machine (SHIMADZU Autograph AGS-X 10 kN). The Vickers hardness and tensile properties were all measured with 3 samples, and the average values were shown with an error range.

## Supplementary Information

Supplementary Information.

## Data Availability

The data that support the findings of this study are available from the corresponding author upon reasonable request.
