# Supplementary material for: Flat hardness distribution in AA6061 joints by linear friction welding
Source: Sci Rep. 2021 Jun 3;11:11756. doi: 10.1038/s41598-021-91249-5 (PMC8175386; doi:10.1038/s41598-021-91249-5)
Supplement: Supplementary file 1 — Supplementary Information. [file 41598_2021_91249_MOESM1_ESM.pdf]

Table 1. Parameters necessary for predicting yield strength of the Al6061 joints obtained at different applied pressures and different regions.

Applied pressure	d (μm)	f	Dislocation density (m^{-2})	S_V ($μm^{-1}$)	θ (°)	D (μm)
Base material	14	2×10^{-2}	3.5×10^{13}	1.3	4.9	45
50 – Center	61	2.1×10^{-4}	7.6×10^{12}	2.1	5.6	2.4
50 – 0.5 mm	26	4.6×10^{-3}	1.38×10^{14}	5.1	5.2	30.9
50 – 2 mm	22	2.1×10^{-2}	3.4×10^{13}	0.7	4.9	59.6
240 – Center	26	6.5×10^{-4}	4×10^{13}	2.3	7	0.3
240 – 0.5 mm	18	5.8×10^{-3}	1×10^{14}	6.6	4.4	39
240 – 2 mm	17	1.8×10^{-2}	4.4×10^{13}	2.9	3.5	35.7

Table 2. Strengthening mechanism of the Al6061 joints obtained at different applied pressures and different regions.

(Unit : MPa)

Applied pressure	Orowan	Bailey	Hall-Petch	Bowen	Predicted strength	Measured strength	Measured hardness
Base material	203.2	67.2	10.4	24.4	355.2	330	115 Hv
50 – Center	4.7	31.3	45.5	74	205.5	240	74 Hv
50 – 0.5 mm	51.4	42.2	12.6	111.9	268.1	-	79 Hv
50 – 2 mm	140.2	66.3	9	40.1	305.9	-	85 Hv
240 – Center	18	71.9	127.8	79.5	347.2	331	119 Hv
240 – 0.5 mm	80.3	113.8	11.2	117.5	372.8	-	113 Hv
240 – 2 mm	158.8	75.3	11.7	69.6	365.4	-	114 Hv

Table 3. Chemical composition of A6061 alloy.

Chemical composition (mass%)								
Material	Mg	Si	Fe	Cu	Cr	Ti	Mn	Al
A6061-T6	0.96	0.59	0.4	0.27	0.12	0.04	0.02	Val.

Table 4. Linear friction welding conditions of this study.

Parameter	Conditions
Applied pressure (MPa)	50, 240
Frequency (Hz)	25
Amplitude (mm)	2
